# Mathematical Modeling of Outdoor Natural Weathering of Polycarbonate: Regional Characteristics of Degradation Behaviors

**DOI:** 10.3390/polym13050820

**Published:** 2021-03-07

**Authors:** Takato Ishida, Ryoma Kitagaki

**Affiliations:** Graduate School of Engineering, Hokkaido University, Nishi-8-chome, Kita-13-jyo, Kita-ku, Sapporo-shi, Hokkaido 060-8628, Japan

**Keywords:** polycarbonate, outdoor aging, degradation rate, climate characteristics

## Abstract

Many natural exposure sites have been developed to ensure the reliability of materials intended for outdoor use. However, the effects of local climate on aging have not been completely understood. This study aimed to elucidate the regional characteristics of natural aging. Non-stabilized and stabilized polycarbonates were monitored in terms of their appearance (yellowing and loss of gloss) during natural weathering at five exposure sites (Tokyo, Kagoshima, Okinawa, Florida, and Arizona) in conjunction with climate fluctuation for up to 24 months. Three approaches were employed to characterize the natural aging behaviors: (i) modeling the rate function of degradation, (ii) evaluating the contribution ratio of individual degradational factors, and (iii) estimating the “synchronicity” by cross-correlation analysis with the climate dataset. The aging rates were the highest in Arizona and lowest in Kagoshima among the five exposure sites. First, prediction curves were constructed from the degradation rate function (variables: UV irradiation, temperature, and humidity), and these curves were found to agree well with the measured aging behaviors. Second, the exposure data in Arizona demonstrated strong temperature dependence, while those in Okinawa and Florida had stronger dependence on UV irradiation compared to other sites. Lastly, the synchronicity between UV irradiation and temperature was the highest in Arizona and lowest in Kagoshima, which can explain the significantly faster deterioration in Arizona and the slow deterioration in Kagoshima.

## 1. Introduction

Polymeric materials suffer from property degradation during outdoor use due to light, temperature, and humidity. Polycarbonate (PC) is a well-known engineering plastic and is widely used in outdoor applications such as buildings, constructions, automobiles, and aircraft. To evaluate the weatherability and lifespan of PC under outdoor exposure, many studies over the past several decades have examined the photo-aging behavior of PC [1,2]. Diepens and Gijsman [1] and Pickett [2] discussed the photochemistry of PC in detail. According to their investigations, degradation of PC starts from the photo-Fries rearrangement reaction and/or photo-oxidation process. The subsequent formation of chromophores and changes in macromolecular architectures (chain scission and crosslinking) should alter the material properties, including the appearance and mechanical characteristics.

Weatherability is one of the most critical aspects of PC for long-term outdoor use. To ensure the material’s reliability, an outdoor exposure test should be conducted. Many exposure sites have been developed over the last several decades. The locations include Okinawa (Japan), Florida and Arizona (United States), the Netherlands, France, Singapore, and Australia [3]. On the other hand, accelerated aging systems were also used for quick assessments of long-term characteristics in order to bring new materials to the market as fast as possible. A variety of artificial exposure test protocols have been developed for demonstrating various environments. Although there are many reports on the ability of artificial exposure tests to reproduce natural exposure effects, some gaps remain between natural and accelerated aging due to additional complexity in the former, such as seasonal fluctuations of climate systems, influences of rainwater, and wetting [4,5].

Polymer degradation behavior is expected to vary somewhat among exposure sites with different climates. Currently, Florida exposure is regarded as the de facto standard [3]. Arizona exposure is also widely used because of the severe environment. However, Arizona is generally much less humid than Florida and has a higher temperature during the summer. Hence, we need a better understanding of the dependence of aging behavior on climate conditions. Such knowledge will help determine the suitable parameters in accelerated aging tests to reproduce the natural degradation behaviors.

Here, we studied the degradation characteristics of non-stabilized/stabilized PC at five exposure sites (Tokyo, Kagoshima, and Okinawa in Japan; Florida and Arizona in the United States). The changes in appearance quality (i.e., yellowing and gloss loss) were analyzed for PC samples during outdoor aging at these sites in combination with the climate conditions. First, we modeled the degradation rate (k) as a function of temperature, ultraviolet (UV) irradiation, and relative humidity (RH). Second, a method was proposed to evaluate the contribution ratio for each of these three variables. Thus, regional characteristics of the exposure sites can be effectively discussed using the above-mentioned natural weathering dataset. Finally, we examined one complex issue in natural aging, namely, the effect of the relative phase between environmental factors. For example, between two sites with the same average UV irradiation and temperature, degradation occurs faster when high UV irradiation coincides with high temperature. The effect of these phase differences can be effectively examined by using cross-correlation analysis [6]. Such an approach enabled us to evaluate the “synchronicity” of climate factors. In particular, the strong synchronicity between UV irradiation and temperature may explain the fast degradation rate in Arizona during summertime.

## 2. Experimental

### 2.1. Samples

Outdoor weathering tests were performed using both non-stabilized and stabilized commercial bisphenol A polycarbonate plates (denoted as PC1 and PC4, respectively), which were kindly provided by Japanese industrial sources. The samples had a dimension of 4 cm × 2 cm × 6 mm and were placed on glass substrates.

### 2.2. Exposure Tests

**Figure 1:**
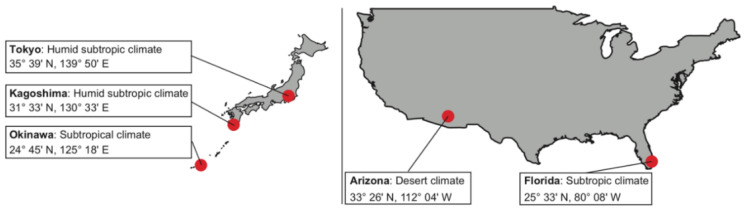
Locations of the five natural exposure sites and their climate classifications.

**Figure 2:**
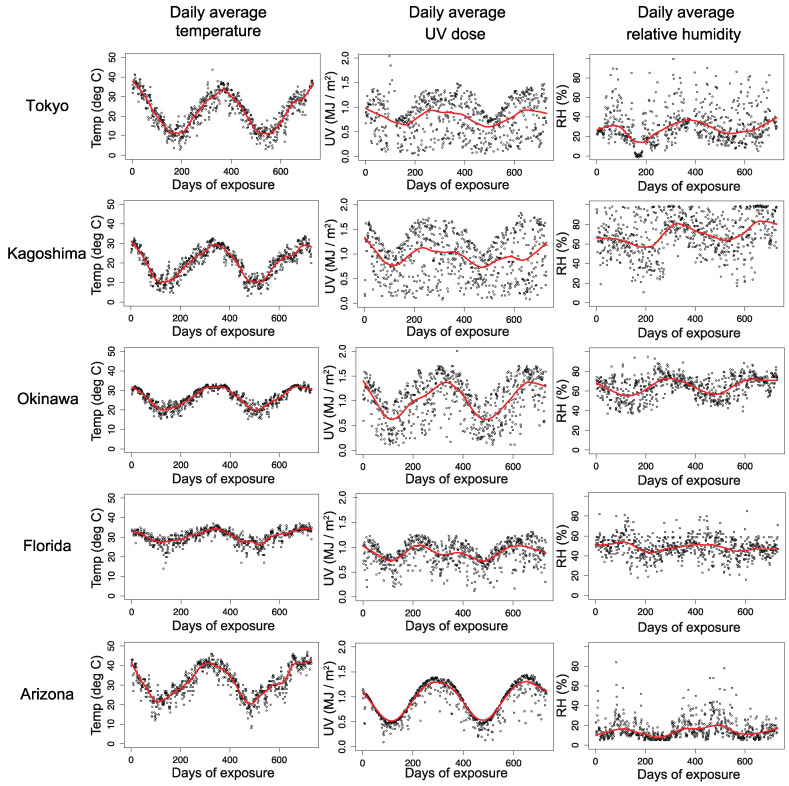
Fluctuations of daily average temperature, UV irradiation, and relative humidity (RH) for five exposure sites. The inserted red lines are shown to guide the eye.

Figure 1 shows the locations of five natural exposure sites and their climate classifications. The daily average temperature, UV irradiation, and RH data are illustrated in Figure 2. The exposed PC samples were analyzed after exposure for 3, 6, 12, 18, and 24 months. Table 1 summarizes the representative climate parameters at the exposure sites.

### 2.3. Color Measurement

Color data were collected using a SUGA TEST color meter. The yellowing index (⊿YI) was based on the CIE standard illuminant D65 and CIE 1931 10° standard observer viewing. Gloss was measured with a Gloss meter UGV 6P (Suga Test Instruments, Tokyo, Japan) at 60°.

## 3. Theory

### 3.1. Modeling of Degradation Rate Function

A variety of approaches have been proposed to estimate degradation rates in the aging process, such as the simple Arrhenius approach [7], the declination angle model proposed by Bauer [8], neural networks [9], a model considering the wetting time [10], and a time–temperature superposition method [11]. In this study, we employed the Striny and Schelling model [12] in combination with Schwarzchild’s law factor [13], as expressed in Equation (1).

(1) $$k\left(I,\;T,\;r.h.\right)=AI^{p}\exp\left(-\frac{E_{a}}{RT}\right)\exp\left(-\frac{\beta }{r.h.}\right)$$

where the pre-exponential factor A is a constant called the frequency factor, T (K) is the absolute temperature, R is the gas constant, I [MJ/m^2^] is the UV irradiation, p is Schwarzchild’s parameter (normally 0<p<1), $E_{a}$ [kJ/mol] is the apparent activation energy, β [%] is the apparent activity parameter for humidity, and r.h.  [%] is the RH. The parameters p, $E_{a}$, and β are determined by fitting to the measured degradational behavior for each material and property. Equation (1) enabled us to consider the effects of temperature, UV irradiation, and humidity on the appearance qualities (i.e., yellowing and gloss loss) in order to understand regional differences in the aging behavior.

### 3.2. Contribution Ratio of Degradational Factors

For a quantitative study of the regional characteristics, we would like to evaluate the contribution ratios of each of the three environmental factors (UV irradiation, temperature, and RH). Since these environmental factors are variables in the rate function k in Equation (1), the total derivative (dk) can be expressed as follows:

(2) $$dk={\left(\frac{\partial k}{\partial I}\right)}_{T,r.h.}dI+{\left(\frac{\partial k}{\partial T}\right)}_{I,r.h.}dT+{\left(\frac{\partial k}{\partial r.h.}\right)}_{I,T}dr.h.$$

Each term on the right side of Equation (2) illustrates the sensitivity of *k* to changes in the corresponding environmental factor (while the other two factors are fixed). Hence, we propose the contribution ratio of each environmental factor as follows:

(3) $$Q_{T}\left[\%\right]=\frac{{\left(\frac{\partial k}{\partial I}\right)}_{T,r.h.}dI}{{\left(\frac{\partial k}{\partial I}\right)}_{T,r.h.}dI+{\left(\frac{\partial k}{\partial T}\right)}_{I,r.h.}dT+{\left(\frac{\partial k}{\partial RH}\right)}_{I,r.h.}dr.h.}\times 100$$

(4) $$Q_{I}\left[\%\right]=\frac{{\left(\frac{\partial k}{\partial T}\right)}_{I,r.h.}dT}{{\left(\frac{\partial k}{\partial I}\right)}_{T,r.h.}dI+{\left(\frac{\partial k}{\partial T}\right)}_{I,r.h.}dT+{\left(\frac{\partial k}{\partial RH}\right)}_{I,r.h.}dr.h.}\times 100$$

(5) $$Q_{RH}\left[\%\right]=\frac{{\left(\frac{\partial k}{\partial RH}\right)}_{I,T}dRH}{{\left(\frac{\partial k}{\partial I}\right)}_{T,r.h.}dI+{\left(\frac{\partial k}{\partial T}\right)}_{I,r.h.}dT+{\left(\frac{\partial k}{\partial RH}\right)}_{I,r.h.}dr.h.}\times 100$$

where $Q_{T},\;Q_{I},\;\mathrm{and}\;Q_{RH}$ [%] are the contribution ratios of temperature, UV irradiation, and RH to material aging, respectively. Using the expression of *k* in Equation (1), the partial derivatives $\left(\frac{\partial k}{\partial \left(\cdot \right)}\right)$ are easily calculated as follows:

(6) $${\left(\frac{\partial k}{\partial I}\right)}_{T,r.h.}=\frac{p}{I}k$$

(7) $${\left(\frac{\partial k}{\partial T}\right)}_{I,r.h.}=\frac{E_{a}}{RT^{2}}k$$

(8) $${\left(\frac{\partial k}{\partial r.h.}\right)}_{I,T}=\frac{\beta }{{\left(r.h.\right)}^{2}}k$$

Because the environmental factors (I, T, and r.h.) do not always fluctuate at comparable magnitudes, dI, dT, and dr.h. can be further expressed in the form of Eq. (9) to consider their variance:

(9) $$dX=\sigma_{X}\times n\;\;\left(X=I,\;T,\;\mathrm{and}\;r.h.\right)$$

where $\sigma_{X}$ is the standard deviation for each environmental factor, and n is an infinitely small number.

### 3.3. Cross-Correlation Analysis

We employed cross-correlation analysis to study the phase difference in the seasonal fluctuations of environmental factors [6]. As a common technique in the field of signal analysis, cross-correlation analysis evaluates the similarity between two different signals with the same periodicity, including their time lag [14]. Cross-correlation is defined as

(10) $$CC\left(\tau \right)=\overset{\infty }{\underset{j=-\infty }{\sum }}F_{1}\left(j\right)F_{2}\left(\tau +j\right)$$

where CC is the cross-correlation function, τ is the time lag, and $F_{1}\;\mathrm{and}\;F_{2}$ are the signal series. In this study, $F_{1}\;\mathrm{and}\;F_{2}$ are two sets of the three sequential environmental datasets (temperature, UV irradiation, and RH).

## 4. Results and Discussion

### 4.1. Environmental Data and Surface Temperature Estimation

**Figure 3:**
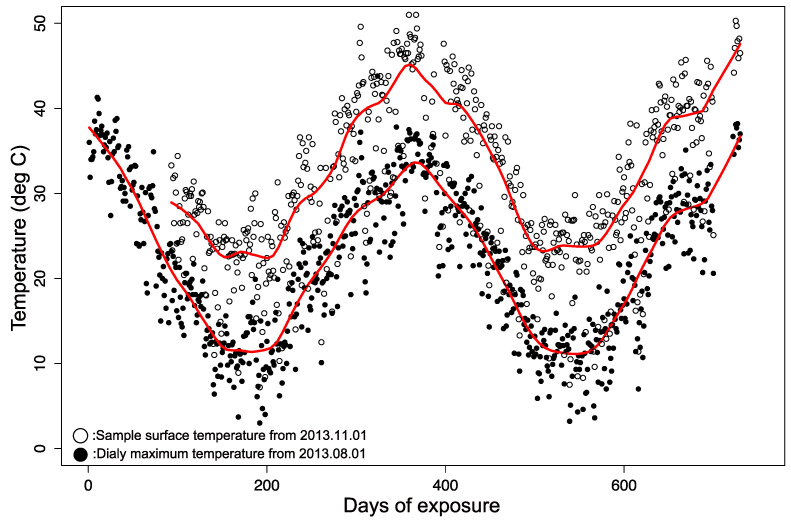
Temperature difference between polycarbonate (PC) surface and air in Tokyo. The red lines are inserted to guide the eye.

In the climate dataset, the UV radiation intensity and temperature should be high, and the humidity should be low during the daytime. Because the PC aging process is possibly initiated by UV, the observed degradation should also occur mainly in the daytime. Hence, we adopted the daily maximum UV irradiation, temperature, and daily minimum relative humidity as the representative daily environmental parameters for reproducing the degradation during the daytime. Additionally, because we focused on the appearance qualities, whose degradation occurs at sample surfaces, the sample surface temperatures should be used as input data for the rate function k. Note that the sample surface temperature tends to be higher than the ambient air temperature due to the absorption of sunlight. To verify their relationship, we measured the surface temperatures of PC samples exposed at the Tokyo site starting on November 01, 2013. As expected, these temperatures show non-negligible differences from the ambient air temperature, as shown in Figure 3.

**Figure 4:**
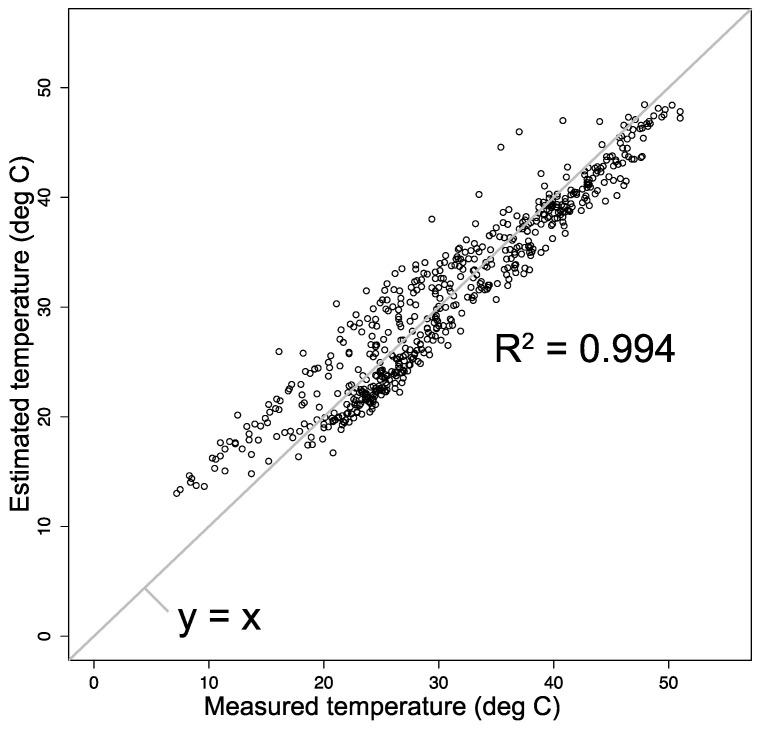
Correlation between measured surface temperature and estimated surface temperature from Equation (11) for the Tokyo data. The two temperatures are strongly correlated (R^2^ = 0.994).

A variety of models have been developed for estimating the difference between ambient temperature and material surface temperature [8,15,16]. Here, we applied a very simple model that assumes the temperature difference is proportional to light intensity, as expressed in Equation (11).

(11) $$T_{mat}=T_{air}+C_{1}I+C_{2}$$

where $T_{mat}$ is the sample surface temperature, $T_{air}$ is the ambient air temperature, and $C_{1}\;\mathrm{and}\;C_{2}$ are constants. Fitting Equation (11) to the data in Figure 3 gave the values of $C_{1}=4.57\;\mathrm{and}\;C_{2}=\;6.78$. Figure 4 demonstrates the very good correlation between the two sets of temperature for Tokyo using Equation (11). Hence, we used the fit to estimate material surface temperatures for all five exposure sites as input data to the degradation rate function.

### 4.2. Outdoor Aging Behavior of Appearance Quality

**Figure 5:**
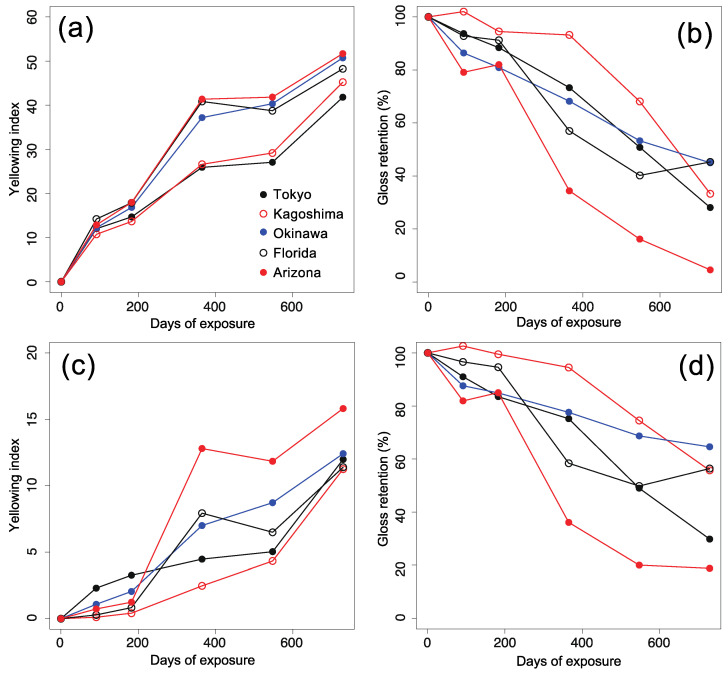
Outdoor aging behavior of appearance quality, (**a**): yellowing for non-stabilized PC samples, (**b**): gloss loss for non-stabilized PC samples, (**c**): yellowing for stabilized PC samples, (**d**): gloss loss for stabilized PC samples. The data are plotted against the integrated UV irradiation for each exposure site (Tokyo, Kagoshima, Okinawa, Florida, and Arizona).

Figure 5 shows the trends of yellowing and gloss loss for non-stabilized and stabilized PC samples. As expected, the yellowing index (⊿YI) increased, and the gloss retention decreased during the degradation process. Yellowing and gloss loss are common phenomena induced by photodegradation. In the literature, the increase in ⊿YI can be attributed to the formation of quinone-type products via photo-Fries reaction and/or the formation of polyconjugated species via photo-oxidation [17,18]. The loss of gloss is possibly due to an increase in surface roughness induced by UV aging [19].

First, we roughly assess the degradation rates at different exposure sites. Although the exact order sometimes depends on the properties of interest (i.e., yellowing or gloss loss) or the samples (i.e., stabilized or non-stabilized), the degradation rates have the following approximate trend:

Kagoshima (slow) < Tokyo, Okinawa, Florida (intermediate) < Arizona (fast)

A direct comparison between the order above and climate parameters in Table 1 suggests that the average maximum temperature (${\bar{T}}_{max}$) of the site could be a principal factor for determining the degradation rates. However, it is difficult to completely describe regional characteristics using a single climate parameter. In fact, the ${\bar{T}}_{max}$ of Arizona does not significantly differ from that of Florida; however, the deterioration of appearance occurs much faster in Arizona than at the other four sites. In addition, the aging rate in Kagoshima is considerably slower than that in Tokyo, even though the two sites have comparable average temperatures. These questions will be discussed in Section 4.5 by using cross-correlation analysis.

The yellowing of stabilized PC samples was somewhat mitigated compared to the non-stabilized ones, suggesting that the stabilizers retard the chemical aging process. On the other hand, the stabilization effect on gloss loss may be less pronounced compared with yellowing. One possible reason is that the loss of gloss could be a rather “physical” phenomenon (e.g., washing out of chemical products by rainwater) accompanied by morphological changes than the “chemical” phenomenon.

### 4.3. Reproducing Outdoor Aging Behavior from the Environmental Dataset

To study the regional characteristics of aging behaviors, we tried to estimate the degradational rate function (k) as formulated in Equation (1) by using the environmental data for each exposure site (Figure 2). We determined the characteristic parameters (p, $E_{a}$, β) that give the best fit to the aging data in Figure 5, using a trial-and-error procedure. Table 2 shows the fitted parameters for each type of sample and property. According to the fitted p values, yellowing of non-stabilized PC had the strongest dependence on UV irradiation compared to other combinations of samples and properties. There is also a certain level of dependence on temperature and very little dependence on humidity. Namely, all β values are very small, and the range of $E_{a}$ is comparable to the literature [20].

The obtained rate functions were able to reproduce the principal aging in the daytime because the main aging mechanism of PC is expected to be photodegradation. Normally, when temperature and UV irradiation are high during the daytime, RH tends to be low. This is the possible reason why the “direct” dependence on RH is quite small.

**Figure 6:**
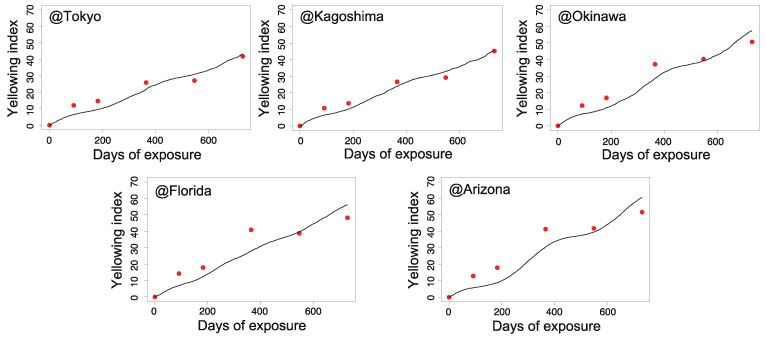
Change in ⊿YI during outdoor aging for non-stabilized PC samples (red dots) and the predicted values (solid lines) for each exposure site (Tokyo, Kagoshima, Okinawa, Florida, and Arizona).

**Figure 7:**
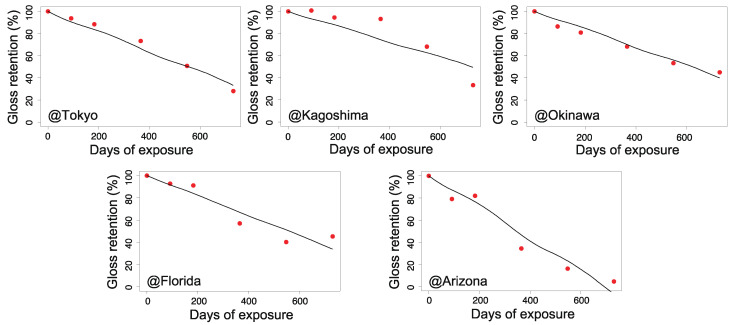
Gloss retention during outdoor aging for non-stabilized PC samples (red dots) and the predicted values (solid lines) for each exposure site (Tokyo, Kagoshima, Okinawa, Florida, and Arizona).

**Figure 8:**
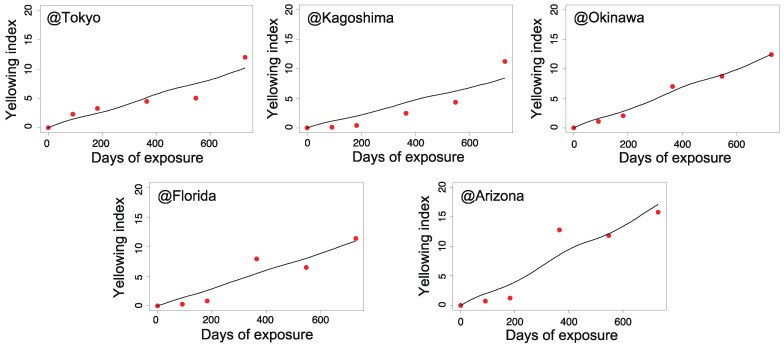
Changes in ⊿YI during outdoor aging for stabilized PC samples (red dots) and the predicted values (solid lines) for each exposure site (Tokyo, Kagoshima, Okinawa, Florida, and Arizona).

**Figure 9:**
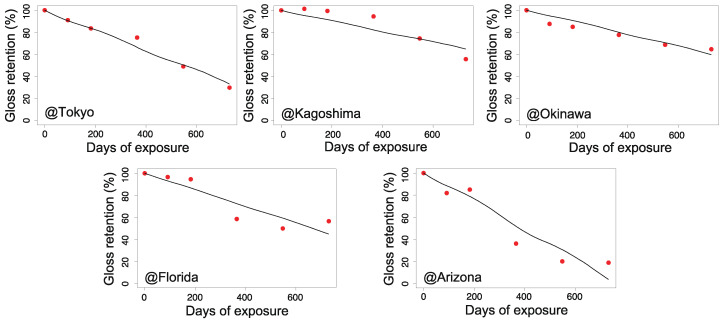
Loss of gloss during outdoor aging for stabilized PC samples (red dots) and the predicted values (solid lines) for each exposure site (Tokyo, Kagoshima, Okinawa, Florida, and Arizona).

Figure 6, Figure 7, Figure 8 and Figure 9 illustrate the validity of the obtained rate functions for each degradation behavior. The curves were produced by integrating the k function, which can be evaluated by sequential substitution of specific environmental factors for each exposure site.

According to Figure 6, Figure 7, Figure 8 and Figure 9, the model can reproduce changes in the appearance of non-stabilized and stabilized PC samples to some degree, including seasonal fluctuations. However, the agreement for stabilized PC samples was somewhat weak at several exposure sites. For instance, ⊿YI and loss of gloss increased quite significantly in Arizona in the period of 6–12 months (corresponding to the summer season). In that period, a certain level of deviations was observed between the data and prediction curves. The results for Kagoshima and Florida also showed similar behaviors in certain periods. This implies that our model cannot completely reproduce the aging behaviors during periods of drastic deterioration. After excluding the periods of intensive degradation, our model could reproduce the measured changes in appearance as a whole. Hence, the fitted parameters in Table 2 are acceptable for reproducing the aging behaviors. In the next section, they will be further analyzed to understand the regional characteristics of natural aging behavior.

### 4.4. Contribution of Climate Conditions

We evaluated the contribution ratios of different environmental factors to the rate function, as described in Section 3.2. In this section, the average values of each exposure site were used to calculate k in Equations (6)–(8), i.e., $k=k\left(\bar{I},\bar{\;T},\bar{\;RH}\right)$. Table 3 illustrates the estimated contribution ratios ($Q_{T}$ and $Q_{I}$) for each combination of sample, property, and exposure site. As expected, the contributions of humidity were all negligible ($Q_{RH}$ < 0.1%) and therefore not shown here. This may originate from a very minor dependence on humidity according to the minuscule β values in Table 2. The effect of temperature may have a greater impact on the loss of gloss than yellowing, and its effect is also stronger for stabilized than non-stabilized PC. Regarding regional characteristics, Okinawa and Florida have similar contribution ratios for both yellowing and gloss loss, where the ratio of UV irradiation is relatively higher than for other exposure sites. On the other hand, Arizona demonstrated a higher contribution from the temperature.

### 4.5. Effects of Phase Between Climate Factors

**Figure 10:**
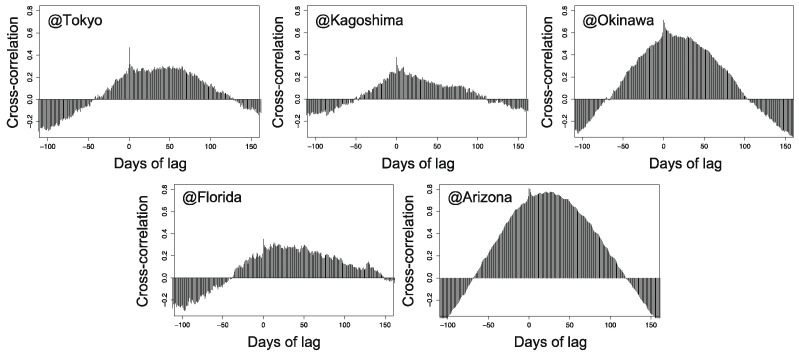
Calculated cross-correlation function between temperature and UV irradiation for each exposure site.

As stated in the introduction, natural aging behaviors cannot be fully explained by the average values of environmental factors (e.g., temperature, UV irradiation, humidity, and rainfall). Here, we discuss the effects of phase between two climate factors using cross-correlation analysis. In Section 4.4, we identified the principal climate factors for PC degradation as temperature and UV irradiation. Thus, we employed temperature and UV irradiation in Equation (10) as $F_{1}\;\mathrm{and}\;F_{2},$ respectively. Figure 10 demonstrates the cross-correlation function between temperature and UV irradiation for each exposure site. The time lag between peak temperature and peak UV irradiation is approximately 20–30 days. At all exposure sites, the peak temperature lagged behind the peak UV irradiation, as shown in Figure 2. We further define “synchronicity” between temperature and UV irradiation as the cross-correlation value at τ=0 in Equation (10), corresponding to the inner product between the two signals without time lag. The order of synchronicity is as follows (evaluated synchronicity values are indicated in brackets):

Kagoshima (0.38) < Florida (0.35) < Tokyo (0.47) < Okinawa (0.72) < Arizona (0.81)

Note that this ranking does not contradict the approximate order in degradation rate discussed in Section 4.2. Hence, the synchronicity between temperature and UV irradiation may determine how the exposure site affects the aging rate, and this may help explain some of the complex aging behavior. Firstly, the very quick deterioration in Arizona could be attributed to the very strong synchronicity between temperature and UV irradiation at that location, although the average temperatures in Arizona are not very different from those in Florida. In contrast, the rate of aging is the slowest in Kagoshima among all exposure sites, despite the similar average temperatures and higher UV irradiation compared to Tokyo. This might also originate from the weaker synchronicity in Kagoshima.

Let us note that the relationship between natural outdoor weathering and accelerated weathering. To date, many accelerated weathering protocols have been proposed to simulate natural weathering, for example, the light/dark cycle and the UV/condensation cycle [3,21]. However, it remains challenging to reproduce the complex behavior of natural aging at actual target sites by using other exposure sites or accelerated aging tests. Proposed synchronicity might be one of the key aspects to solve such a complexity. To better emulate the complex natural aging, we will try to introduce synchronicity in future studies for a quantitative forecasting model of degradation rate. In addition, here, we only considered the daily average of climate data, whereas the intra-day fluctuations of the environment might also have significant influences. For example, the intra-day temperature fluctuation is quite large in Arizona. Therefore, it is worth further improving our model by using more fine-grained (e.g., hourly) data.

## 5. Conclusions

We obtained outdoor weathering data, namely yellowing (YI) and loss of gloss, for non-stabilized and stabilized PC samples at five exposure sites in Japan (Tokyo, Kagoshima, and Okinawa) and the United States (Florida and Arizona) in order to examine the regional characteristics of different sites. As expected, YI values increased, and gloss retentions decreased throughout the degradation process. Among the five exposure sites, the aging rates were obviously the highest in Arizona and lowest in Kagoshima. The order of aging rate seems to be related to the average maximum temperature (${\bar{T}}_{max}$) of exposure sites; however, the latter does not completely explain the exposure site dependence.

Several analyses were employed to obtain deep insights into the climate factors that affect PC aging. First, a model for the degradation rate function (k) was constructed by combining the temperature and humidity dependence in the Striny and Schelling model and Schwarzchild’s law factor for the contribution of UV irradiation. We investigated the validity of the obtained k for each combination of sample, property, and exposure site. The prediction curves constructed from the model seem to follow the actual aging data. Second, we evaluated the contribution ratios of three environmental factors by taking the total derivative of the rate (dk). For the regional characteristics, Okinawa and Florida have similar contribution ratios for both yellowing and gloss loss, where the ratio of UV irradiation is higher than at other exposure sites. Hence, Okinawa might be a possible alternative to Florida for outdoor exposure testing. On the other hand, Arizona demonstrated a higher contribution ratio from temperature. In addition, the impact was greater on the loss of gloss than yellowing and greater for stabilized than non-stabilized PC. Such analysis can shed light on the regional characteristics of aging behavior and the dominant climate factor. It was also revealed that relative humidity only makes a very small “direct” contribution for all exposure sites compared to UV irradiation and temperature. However, the effect of humidity may be implicitly included in the contributions from the other two climate factors. Future research should try to decouple the effects of UV irradiation and temperature from that of humidity.

Finally, cross-correlation analysis was used to reveal the phase difference between temperature and UV irradiation across the exposure sites. The obtained synchronicity between the two sets of signals has the same order among the sites as the rate of aging. The synchronicity can also explain the much faster deterioration rate of PC appearance in Arizona and the slowest deterioration in Kagoshima, for which the evaluated synchronicity between UV irradiation and the temperature is the highest and lowest respectively.

## Figures and Tables

**Table 1:** Summary of representative climate parameters in outdoor aging. $\bar{T}$ is the average ambient temperature, ${\bar{T}}_{max}$ is the average maximum ambient temperature, ${\bar{T}}_{min}$ is the average minimum ambient temperature, $\bar{I}$ is the average UV irradiation (300 to 400 nm), ($\bar{RH}$ ) is the average relative humidity, ($\bar{BPT\;}$ ) is the average black panel temperature, ($\bar{Rf\;}$ ) is the average rainfall, and θ is a sample mounting angle facing south.

Location	Duration	$\bar{T}\;({}^{\circ}C)$	${\bar{T}}_{max}\;({}^{\circ}C)$	${\bar{T}}_{min}\;({}^{\circ}C)$	$\bar{I}$ (MJ/m^2^⋅year)	$\bar{RH}\;(\%)$	$\bar{BPT\;}({}^{\circ}C)$	$\bar{Rf\;}(\mathrm{mm}/\mathrm{year})$	θ [°]
Tokyo	Aug. 2013 ~ July 2015	16.6	21.7	11.8	0.77	53.6	26.2	1876	45
Kagoshima		15.9	20.0	12.8	0.94	87.0	18.5	2791	30
Okinawa		23.3	26.3	20.9	1.00	77.2	27.6	1577	20
Florida	Sep. 2013 ~ Aug. 2015	24.5	30.4	19.6	0.87	74.1	28.1	3876	26
Arizona		22.7	31.3	14.0	0.90	33.1	23.9	237.0	45

**Table 2:** Fitted characteristic parameters for each type of sample and property. p is dimensionless.

Property	Sample	p	$E_{a}\;(\mathrm{kJ}/\mathrm{mol})$	β (%)
⊿YI	PC1	0.99	18.06	$1.05\times 10^{-6}$
	PC4	0.32	10.70	$1.33\times 10^{-6}$
Gloss retention	PC1	0.16	10.94	$1.00\times 10^{-6}$
	PC4	$1.01\times 10^{-6}$	10.71	0.05

**Table 3:** Contribution ratios ($Q_{I}$ and $Q_{T}$ (%)) for each combination of sample, property, and exposure site.

**Non-stabilized,** **⊿YI**	**$Q_{I}\;\left(\%\right)$**	**$Q_{T}\;\left(\%\right)$**	**Stabilized,** **⊿YI**	**$Q_{I}\;\left(\%\right)$**	**$Q_{T}\;\left(\%\right)$**
Tokyo	67.2	32.8	Tokyo	53.1	46.9
Kagoshima	70.2	29.8	Kagoshima	53.6	43.4
Okinawa	74.8	25.2	Okinawa	62.2	37.8
Florida	74.9	25.1	Florida	62.4	37.6
Arizona	64.3	35.7	Arizona	49.9	50.1
**Non-stabilized, Gloss loss**	**$Q_{I}\;\left(\%\right)$**	**$Q_{T}\;\left(\%\right)$**	**Stabilized, Gloss loss**	**$Q_{I}\;\left(\%\right)$**	**$Q_{T}\;\left(\%\right)$**
Tokyo	67.2	32.8	Tokyo	53.1	46.9
Kagoshima	70.2	29.8	Kagoshima	53.6	43.4
Okinawa	74.8	25.2	Okinawa	62.2	37.8
Florida	74.9	25.1	Florida	62.4	37.6
Arizona	64.3	35.7	Arizona	49.9	50.1

## Data Availability

The data that support the findings of this study are available on request from the corresponding author. The data are not publicly available due to industrial confidential.
